# Endothelial leak and knowledge gaps: yellow fever virus non-structural protein 1 and the antibodies that bind it

**DOI:** 10.1186/s12985-025-02955-9

**Published:** 2025-10-21

**Authors:** Samantha R. Osman, William B. Messer

**Affiliations:** 1 Molecular Microbiology and Immunology Department, Oregon Health & Science University, Portland, OR USA; 2 Department of Medicine, Division of Infectious Disease, Oregon Health & Science University, Portland, OR USA

**Keywords:** Orthoflavivirus, Yellow fever virus, Non-structural protein 1, Antibodies, Yellow fever 17D vaccine

## Abstract

Yellow fever virus, family Orthoflaviviridae, is the causative agent of yellow fever – a lethal mosquito-borne disease endemic to South America and Africa. Recent advances in understanding the mechanism of orthoflavivirus pathogenesis, particularly for dengue (DENV), demonstrate a role for the secreted viral non-structural protein 1 (NS1) in both animal and human models. The NS1 protein’s most well described pathogenic mechanism is its ability to induce vascular endothelial permeability in a tissue organ specific manner, contributing to orthoflavivirus disease severity. Surprisingly, there is minimal research published on the role of NS1 in YFV pathogenesis, despite the clear and present danger of YFV outbreaks in endemic countries. Understanding the role of NS1 in YFV pathogenesis is critical for the development of therapeutic interventions. Notably, while vaccination efforts for orthoflaviviruses have been historically difficult, the live attenuated vaccine (YFV-17D) against YFV has been hailed as not only the most successful orthoflavivirus vaccine but is among the most successful live virus vaccines ever created. Despite YFV-17D’s widespread use since its introduction in the 1930’s, the best described mechanism of protection is the vaccine’s potent neutralizing antibodies. Our knowledge of NS1 pathogenic mechanisms has expanded since the development of YFV-17D, but how vaccine mediated immunity may interact with NS1 during natural YFV infection is not well described. In this review we describe the current knowledge of YFV NS1 mediated pathogenesis and adaptive immunity directed against YFV NS1 in a variety of animal and human models.

**Graphical Abstract:**

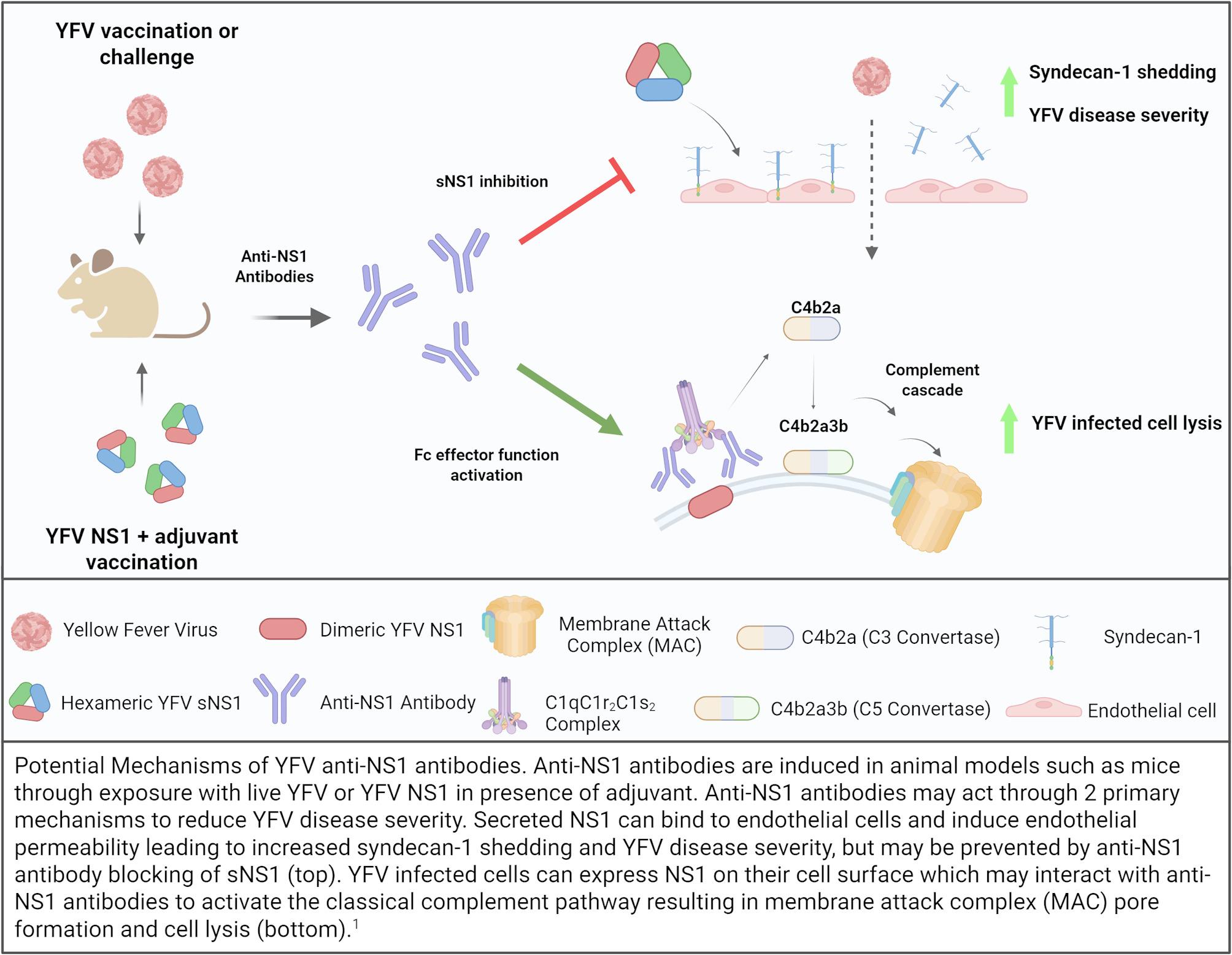

**Supplementary Information:**

The online version contains supplementary material available at 10.1186/s12985-025-02955-9.

## Introduction

**Fig. 1:**
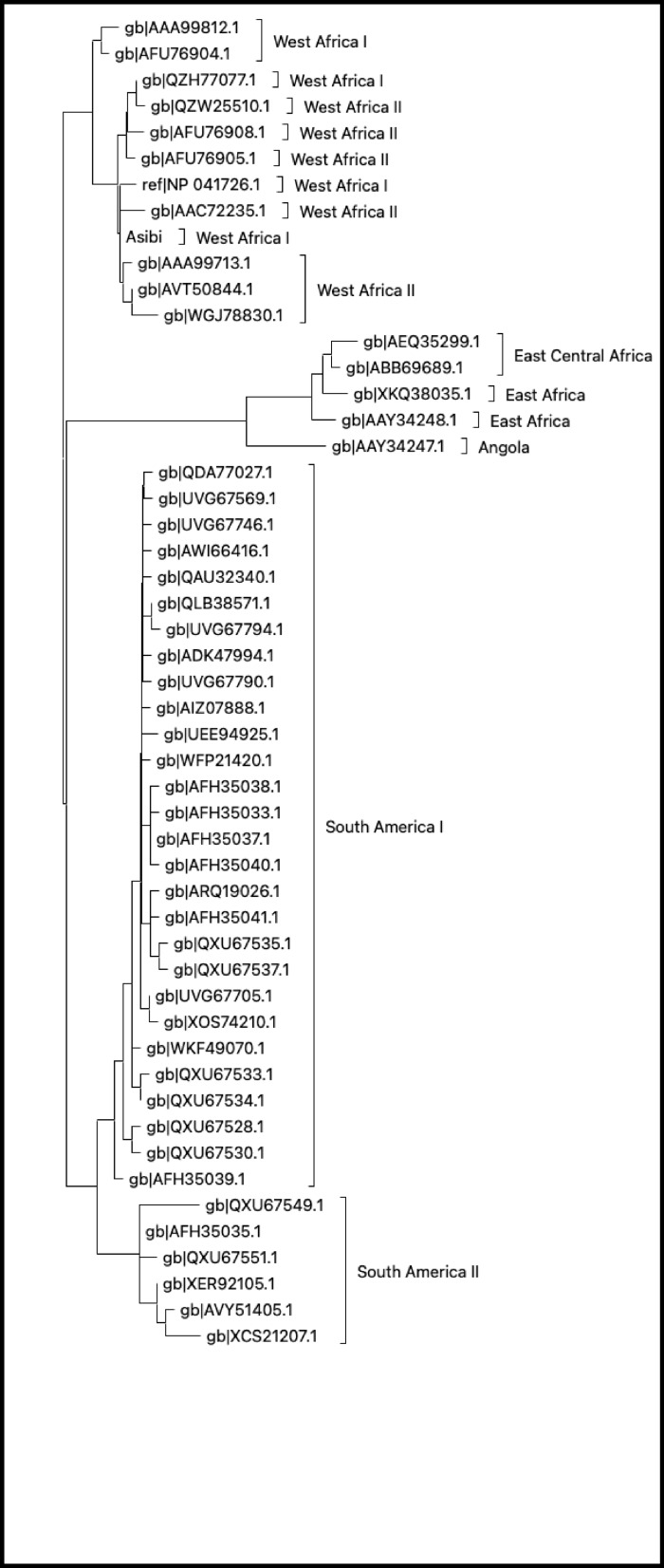
Maximum likelihood tree showing the phylogenetic relationship of 51 wild-type YFV NS1 proteins by amino acid sequence. Tree was constructed and visualized using MEGAX [13]. List of YFV sequences used in supplementary Table 1

Yellow fever virus (YFV) is a mosquito transmitted virus that the World Health Organization (WHO) estimates cause 200,000 infections per year and 30,000 deaths worldwide [2]. YFV is a single stranded positive sense RNA virus of the Orthoflaviviridae genus and is related to other clinically relevant orthoflaviviruses including the dengue viruses (DENVs), Zika virus (ZIKV), West Nile Virus (WNV), and Japanese Encephalitis Virus (JEV). All orthoflavivirus genomes encode 3 structural proteins (Capsid, pre-Membrane, and Envelope) along with 7 non-structural proteins (NS1, NS2A, NS2B, NS3, NS4A, NS4B, and NS5) [3]. YFV is transmitted by mosquito vectors in sylvatic non-human primate transmission cycles, primarily by the *Haemagogus* and *Aedes* genus [4, 5]. YFV is endemic in the tropical and sub-tropical regions of Africa and Central/South America and there are 7 YFV genotypes: West Africa I/II, East Africa, East Central Africa, Angola, and South America I/II (Fig. 1). In 2023, the WHO estimates there are 34 African countries and 13 Central/South American countries where YFV transmission occurs, putting millions at risk of yellow fever infection [2]. Even with the introduction of the highly successful vaccine YFV-17D, YFV outbreaks still occur, making this disease an ever-persistent threat to human health [6]. More research is needed to better our understanding of YFV mechanisms of pathogenesis, of which, the NS1 protein has gained particular interest within the past 20 years. The NS1 protein, in addition to its role in viral genome replication, is secreted from infected cells and its best described function is (its ability) to induce endothelial permeability [7, 8]. Animal studies across the different orthoflaviviruses have demonstrated varying degrees of protection mediated by antibodies directed against NS1, further highlighting the potential role that NS1 plays in disease progression [9–12]. This review will further examine what is known about YFV NS1 pathogenicity and the evidence that humoral immunity against YFV NS1 may be important against severe disease outcomes.

## Yellow fever disease & YFV-17D vaccination

YFV disease symptoms occur within 3–6 days of infection and include fever, headaches, malaise, nausea, and vomiting [14]. Infected individuals may clear the infection or go into a brief remission followed by an intense “intoxication” phase of the disease characterized by severe multi-organ dysfunction, with the liver as the site of viral replication and tissue damage. Patient mortality at this stage ranges from 20 to 60% and is associated with elevated viral load, neutrophil counts (>4000 cells/mL), AST levels, cytokines (IL-6, TNFα, IFNγ, IL-17, IL-1β, IL-5), and chemokines (CXCL10, CXCL8, CCL2, CCL4, CCL5, FGF-basic, G-CSF, and GM-CSF) [15–18] Histopathological analysis of livers of fatal YFV cases display extensive hepatocyte apoptosis and steatosis primarily in the midzonal region along with liver vascular damage [19, 20].

Although there are currently no specific antivirals for yellow fever, prevention is possible through mosquito control efforts and administration of the widely used live attenuated 17D vaccine strain. The YFV-17D strain was created in the 1930’s through serial passaging of the West African I genotype Asibi strain of YFV through mouse and chick brain and chick embryonic tissues. There are only 32 amino acid changes between the virulent and live attenuated strains, primarily in the Envelope and NS2a gene and only 2 mutations in the NS1 gene [21–23]. This original YFV-17D strain is used to generate the two primary substrains used for vaccine production, 17D-204 (manufactured in the United States, the United Kingdom, France, Colombia, Russia, Senegal, and Switzerland) and 17DD (manufactured in Brazil) [24]. Several studies have highlighted the robust induction of neutralizing antibodies (NAbs) in YFV-17D vaccinees that last several years and are often attributed as the primary source of protection from YFV infection [25–32]. However, most studies on YFV-17D elicited NAbs use only the YFV-17D strain to determine neutralizing potency – and the true breadth of these NAbs against any of the other 6 YFV genotypes has not been fully described. This concern of NAb breadth is highlighted in work by Haslwanter et al. 2022 that found reduced neutralizing potency of YFV-17D elicited NAbs (*n* = 16 US vaccinees) against a recent 2017 Brazilian YFV strain used in a reporter virus particle system, due to alterations in domain II of the Envelope protein [33]. These results have been further validated by Goncalves et al. 2024 using a YFV isolate from the 2017–2018 Minas Gerais, Brazil outbreak against YFV-17D vaccinee serum (*n* = 23) which also demonstrated reduced neutralizing capacity [34]. As more outbreaks of YFV occur it is important that we further our understanding of the potential limitations of NAbs as mutations in the Envelope protein may promote viral escape and identify potential alternative mechanisms of protection that may be conferred by the vaccine. As described below, there is increasing evidence of the potential role of NS1 in YFV disease severity, and thus more work is needed to further our understanding of immunological responses post-vaccination directed against YFV NS1 which could promote protection.

### Non-structural protein 1 (NS1) structure

Non-structural protein 1 (NS1), one of five non-structural genes encoded by the orthoflavivirus genome, has garnered particular attention for other orthoflaviviruses for its unique role in pathogenesis. The orthoflavivirus NS1 monomer (positions 779–1187 in YFV polyprotein) has a mass of 45–55 kDa dependent on its glycosylation state and consists of 3 domains: β-roll (amino acids 1–29), wing (amino acids 30–180), and β-ladder (amino acids 181–352) [35]. The β-roll contains β-hairpins that stabilize the dimer form of NS1. The wing and β-ladder domains have N-linked glycosylation site at Asn-130 and Asn-208 for YFV [36]. These glycosylation sites are important for NS1 secretion, viral replication, and disease severity for YFV and other orthoflaviviruses [37, 38].

**Fig. 2:**
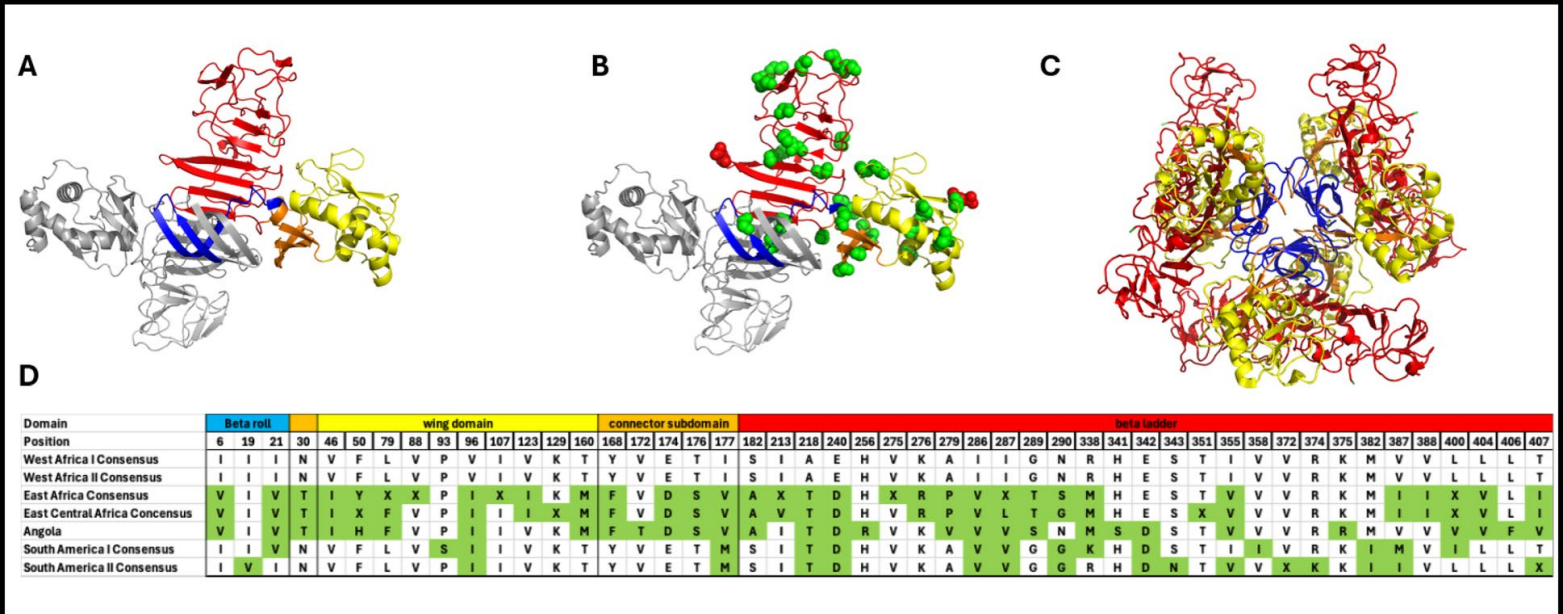
(**A**) Cartoon structure of YFV non-structural protein 1 dimer structure showing one subunit in grey and one subunit colored by domain: β-roll (blue), wing domain (yellow), wing sub-domain connector (orange), central β-ladder (red). N-linked glycosylation sites are denoted by red spheres. Image structure predicted in AlphaFold 3 (https://alphafoldserver.com/) and visualized using Pymol V2.5.4 (Schrondinger, LLC). (**B**) Green spheres indicate locations on the NS1 monomer of amino acid that vary between yellow fever virus NS1 proteins (also summarized Fig. 1D). (**C**) NS1 hexamer showing the arrangement of three NS1 dimers in which the 3 β-rolls (blue) face the interior of the hexamer, creating a hydrophobic pocket capable of fitting lipid cargos in the center of the hexamer. (**D**) Alignment of consensus sequences across the seven YFV genotypes by NS1 domain and amino acid position. Residues that vary are highlighted in green. “X” denotes more than one residue polymorphism at that site for that genotype (see supplemental table S2)

Orthoflavivirus NS1 forms higher order dimeric, tetramer, and hexameric structures (Fig. 2A, B, and C) [39, 40]. The higher order NS1 forms require binding of the β-roll domains between monomers and through hydrophobic interactions [40]. The larger hexameric form of NS1 consists of 3 dimers where the β-rolls create the inner core that is reported to contain lipids. Lipid studies of DENV NS1 have shown that triglycerides, mono/diacylglycerols, cholesteryl esters, and phospholipids are associated with this region of the viral protein [41]. The YFV NS1 dimer is also found associated with cellular membranes, however, the role this surface associated NS1 plays is currently unknown [42].

**Fig. 3:**
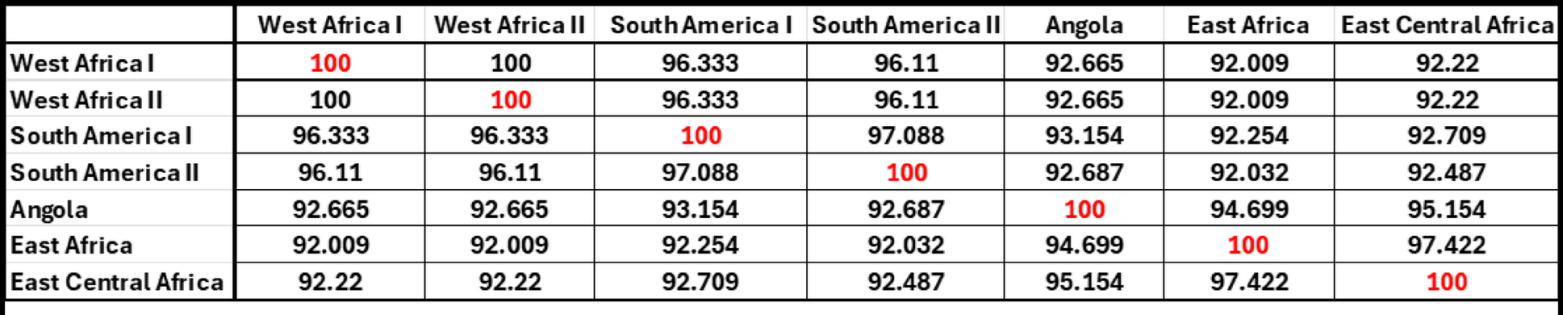
Identity matrix using YFV genotype consensus sequences. Pairwise comparisons of consensus sequences for each YFV genotype generated using Genious

All YFV genomes encode NS1, which is required for viral replication. The NS1 protein has a high degree of amino acid conservation among the 7 YFV genotypes: West Africa I/II, East Africa, East Central Africa, Angola, and South America I/II as seen in Figs. 2D and 3. As shown in Fig. 2D and in supplementary Table 2, most amino acid differences are within the β-ladder domain (across the domain there is 22% amino acid variation) compared to the β-roll (17%) and wing (15%) domains. Amino acid conservation in the β-roll domain across these NS1 protein sequences may be due to this domain’s role in forming the higher order dimer and hexameric structures of NS1. Figure 3 displays the pairwise consensus sequence alignment, we see that overall YFV NS1 demonstrates 92.009% – 100% homology across all genotypes, making it a highly conserved protein among YFV strains.

There is limited NS1 sequence homology between YFV and other orthoflaviviruses, with some studies demonstrating only 42–47% similarity across selected reference strains [43]. Thus, it is unclear the degree to which studies of other orthoflavivirus NS1 mechanistic studies may or may not recapitulate the functions of YFV NS1.

### NS1’s role in the orthoflavivirus life cycle

On infection, YFV virions bind to unknown host receptors and enter the cell via clathrin mediated endocytosis where endosomal acidification results in membrane fusion and uncoating to release the + ssRNA genome into the cytosol [44]. The genome is translated by the host cell’s ribosomes to create a single polyprotein that contains both structural and non-structural proteins. This polyprotein is translated at the endoplasmic reticulum and is then cleaved by NS3 or host proteases into their respective viral proteins [45]. The other NS proteins include: NS2A which binds to the 3’ UTR of the viral RNA genome, NS2B acts as a cofactor for NS3, NS3 has multifunctional enzymatic activities (protease/phosphatase/helicase), NS4A/B proteins are hydrophobic and embedded in the membrane and are also involved in membrane curvature, and NS5 is the largest protein that contains an RNA dependent RNA polymerase domain [46]. Together, expression of these NS1 proteins alone can facilitate ER membrane curvature to form replication compartments, as demonstrated with ZIKV NS proteins in HeLa cells [47, 48]. These distinct compartments facilitate YFV replication by concentrating the host and viral components required for replication and preventing host cell detection by immune sensors [49, 50].

In one study using alanine scanning mutagenesis, a temperature sensitive YFV NS1 mutant (termed ts25 with alanine mutations at K-296 and R-299, both in the β-ladder domain) was isolated that produced more viral plaques at 32 °C than 39 °C [51]. Infecting SW-3 cells (adrenal cortex carcinoma derived) at 39 °C with the ts25 mutant demonstrated undetectable positive strand RNA, indicating that ts25 affects early RNA accumulation. They performed temperature shift experiments – growing ts25 at the permissive 32 °C for 45 h then washing cells and shifting to 39 °C for 3 h – and saw similar viral titers compared to ts25 that was grown at 32 °C the entire time. This indicates the ts25 mutant does not affect viral release and is involved in the early stages of viral replication. In another study, complementation of a noncytopathic Sindbis virus vector encoding full length YFV NS1 with a YFV-17D clone lacking NS1 restored replication of the virus, further indicating that NS1 is required for YFV replication [52]. Work by Scaturro *et al.* 2015 demonstrate that 48 h after infection in Vero cells with HA tagged DENV NS1, in cell lysates the NS1 immunoprecipitated with the structural proteins (Envelope and precursor Membrane) indicating that it could potentially facilitate virion formation at the ER, similar to other orthoflaviviruses [53].

### Surface bound YFV NS1

In addition to NS1’s role at the ER in the formation of viral replication compartments, the YFV NS1 dimer is detected on the surface of infected host cells. As demonstrated by Despres *et al.* 1991, 24 h after infection of insect *S. frugiperda* (Sf9, armyworm) cells with their baculovirus vector containing YFV Env and NS1 genes they were able to fluorescently label the surface of cells using anti-NS1 monoclonal antibodies (mAbs) [54]. Interestingly, they were unable to label the surface of infected cells using anti-Env mAbs, indicating that their recombinant Env was not expressed at the cell surface. These results resemble the findings by Schlesinger *et al.* 1990 that 24 h after YFV-17D infection of SW13 cells there was significantly higher anti-NS1 antibody binding to the surface of cells compared to anti-Env antibody [42]. Together, these results indicate that YFV NS1 can be trafficked to the cell surface when expressed alone or during YFV infection. The exact mechanism of NS1 dimer surface expression of orthoflavivirus infected cells is not well understood, but some studies using DENV suggest that NS1 binds to the plasma membrane through a GPI-anchor that is post-translationally added to the NS1 and may associate with lipid rafts [55, 56]. However, the function of this surface bound NS1 for orthoflaviviruses is unknown.

### Secreted NS1 hexamer

In addition to the surface dimer, NS1 can be trafficked through the Golgi to undergo N-linked glycosylation at its 2 glycosylation sites (Asn-130 and Asn-208) and form a hexamer which is then secreted from the cell (sNS1) [8]. sNS1 binding to the plasma membranes of uninfected cells through interactions with surface GAGs, particularly on endothelial cells, has been demonstrated with DENV sNS1 [57]. In vitro evidence of NS1 secretion after infection with the vaccine YFV-17D strain has been shown in Vero and Huh7.5 cell supernatants via ELISA and Western blot [58, 59]. YFV NS1 is detectable in cell lysates as soon as 48 h post-infection and in the supernatant in appreciable quantities 72 h post-infection, with a steady state of sNS1 thereafter. This suggests that, like other orthoflaviviruses, YFV sNS1 is secreted during infection and could play a similar role in mediating pathogenicity as seen with DENV.

### NS1’s role in vector competence

The role and importance of NS1 in the arthropod vectors for orthoflavivirus replication and transmission is not well described for any of the orthoflaviviruses. In the vector, midgut escape by viruses ingested in the bloodmeal is vital for viral dissemination to the salivary glands where it replicates and can be transmitted [60]. Interestingly, work by Liu *et al.* 2016 demonstrated that DENV2 NS1 presence in bloodmeals enhanced viral acquisition by *Aedes aegypti* mosquitoes [61]. After feeding mosquitoes with NS1 alone, the RNA-seq analysis of midguts demonstrated decreased mRNA expression of genes relating to reactive oxygen species (ROS) production and JAK-STAT signaling. ROS and JAK-STAT are important components of mosquito innate immunity that have been shown to help mount responses against *Plasmodium spp.* and bacterial infections [62]. Notably, JAK-STAT signaling is highly conserved between vertebrates and mosquitoes [63]. Thus, downregulation of these mosquito immune responses may help facilitate YFV infection in the midgut and spread to the salivary glands for transmission.

While YFV NS1 is detectable in midgut lysates as a measure of viral replication at 7- and 14-days post-infection with YFV-Dakar HD 1279 strain in *Aedes aegypti*, the protein was measured only in the monomeric and dimeric form, so it is unclear whether NS1 is also secreted in the higher order hexamer form in the midgut [64]. This study also detected small amounts of monomer/dimer YFV NS1 in the salivary gland lysates only at 14-days post-infection. The mechanisms of YFV NS1 in mediating midgut escape are currently unknown.

Several studies have shown YFV-17D has a limited ability to infect and disseminate in *Aedes aegypti*. This low infectivity has been primarily attributed to reductions in viral entry into the midgut mediated by mutations in the Envelope protein [65, 66]. The midgut barrier hypothesis is further supported by the restoration of YFV-17D replication and dissemination to the legs and salivary glands 10 days after infection via intra-thoracic inoculation which bypasses the midgut [64]. Aside from the Envelope protein, there is some evidence that YFV NS1 may be important for viral dissemination in the mosquito. In a study by Amraoui *et al.* 2018 they serially passaged a South American I genotype YFV strain (YFV-74018) between either two *Aedes albopictus* colonies collected from Manaus or Rio de Janeiro (PNMI) and C6/36 cell lines to determine genetic changes in virus adaptation to these mosquito vectors that while present in YFV at risk areas are not a common vector of YFV disease [67]. Mosquitoes were infected and after 21 days either the head (used for early passages since virus was undetectable in saliva) or saliva were collected and pooled for infection on C6/36 cells – supernatant derived from this propagation was then used to infect the next round of mosquitoes. YFV-74,018 became detectable in the saliva of both mosquito colonies after 10 passages indicating potential transmission adaptations and virus genome sequencing revealed a fixed non-synonymous substitution in the NS1 gene wing and β-ladder domains (I2772T in Manaus and S3303N in PNMI mosquitoes) and a synonymous substitution in the E gene (A1702G in PNMI). This work indicates that the YFV NS1 protein could influence vector competence, however, more work is needed to fully understand these mechanisms.

### NS1’s role in pathogenesis

NS1 has been an especially important viral protein due to its unique role in pathogenesis. This has been most comprehensively described for DENV NS1 and its ability to induce endothelial cell permeability, although a similar function in an endothelial cell specific manner has been described for YFV, ZIKV, and WNV. Endothelial cells form a single cell layer along the luminal side of blood and lymphatic vessels that help regulate blood flow and immune cell trafficking, with tissue specific phenotypes depending on the organ [68]. These endothelial cells are covered with a carbohydrate rich layer called the glycocalyx that is comprised primarily of proteoglycans and glycoproteins [69]. The glycocalyx under homeostatic conditions protects the thin endothelial layer from the shear stress of blood flow, repels blood cells and platelets, and helps regulate vascular permeability [70].

Soluble DENV NS1 (monomer, dimer, and hexameric forms present) has been shown to attach to endothelial cells specifically through interactions with the surface glycosaminoglycans (GAGS), the glycan component of proteoglycans, particularly heparan sulfate and chondroitin sulfate E [57]. The bound DENV NS1 is then endocytosed into endothelial cells which results in upregulation of sialidases and heparinase resulting in endothelial glycocalyx disruption [7, 71]. This glycocalyx disruption is associated with the plasma leakage seen in severe DENV infections [72]. Another study by Modhiran *et al.* 2015 suggests that DENV NS1 can activate TLR4 on immune cells to release pro-inflammatory cytokines and endothelial cells to induce permeability, evident by the reduction in human microvasculature endothelial cell leakage when pretreated with an anti-TLR4 agonist prior to DENV NS1 addition [73]. Markers of endothelial glycocalyx dysfunction for DENV include measuring plasma levels of hyaluronan, heparan sulfate, chondroitin sulfate, and syndecan-1 [74–76]. DENV NS1 has been correlated with viremia, further implicating the role of NS1 and disease severity [77].

While research on YFV NS1’s role in pathogenesis is quite limited, there is evidence that YFV NS1 also induces endothelial permeability. A study by Puerta-Guardo *et al.* 2019 demonstrated that YFV-17D derived NS1 induced vascular endothelial cell permeability in vitro as measured through decreased trans-endothelial electrical resistance (TEER) after addition of NS1 to primary human liver sinusoidal endothelial cells (HLSECs) [78]. These YFV-17D treated HLSECs also displayed reduced levels of surface sialic acid and heparan sulfate and elevated cathepsin-L and heparinase expression. In this same study, administration of the YFV-17D NS1 to C57BL/6 mice followed by Evans blue dye resulted in increased dye present in the liver, indicating tissue level leakage. Together their data suggests that YFV NS1 preferentially promotes endothelial permeability in the liver, which is the primary organ affected during YFV infection. Thus, this indicates that YFV derived NS1 could influence the disease severity of YFV infections.

A few studies have looked at sNS1 during natural YFV infections, including work by Ricciardi-Jorge *et al.* 2017 that first demonstrated that YFV sNS1 could be quantified in ranges of 284–4,597 ng/mL in the serum of acutely infected YFV individuals, which is within the range of previously described acute DENV2 sNS1 in plasma (600–15,000 ng/mL) [59, 77]. Unfortunately, in this study the sample size was small (*n* = 15) and there was no further analysis of relationships between YFV sNS1 and viremia or disease outcomes. More recently, work by de Sousa *et al.* 2024 demonstrate elevated serum sNS1 and syndecan-1 in severe (*n* = 39) compared to non-severe YFV infections (*n* = 11) in a Brazilian cohort [79]. YFV sNS1 correlated highly with syndecan-1 levels, neutrophil count, hematocrit, and indirect bilirubin. In this study syndecan-1 (which is a marker of endothelial permeability) correlated highly with YFV sNS1, sex (male), hospitalization, viral load, liver impairment (AST, ALT, total/direct/indirect bilirubin), kidney impairment (creatinine), coagulopathy, and death. These studies demonstrate the potential importance of sNS1 effects during YFV infection.

### YFV-17D vaccination derived anti-NS1 antibodies in humans

Since YFV-17D is a live attenuated strain that actively replicates after vaccination, it would be expected that NS1 is expressed following vaccination, eliciting an NS1 specific immune response. However, despite nearly a century of use, there are surprisingly few studies that have measured the presence of anti-NS1 antibodies after YFV-17D vaccination. In a study by Raulino *et al.* 2021 that sought to demonstrate the feasibility of a multiplex NS1 antibody specific Luminex platform for serological screening of non-human primates (NHPs), they used YFV-17D vaccinees (*n* = 18; participants from Belgium, Colombia, and the Democratic Republic of the Congo) as their positive control for the specificity and sensitivity analysis [80]. Interestingly, the YFV-17D NS1 antigen had lower specificity (93%) and sensitivity (44%) compared to the other orthoflavivirus NS1 antigens in this assay. However, no further information was included in this study on the demographics of the YFV-17D vaccinees that may have affected the anti-NS1 antibody levels such as time post-vaccination.

In another study by Chen *et al.* 2024, they demonstrated via Western blot that anti-NS1 antibodies were detectable in vaccinees that were < 1 month to 5 years post-vaccination (*n* = 19; participants from the United States and Brazil) and in YFV-17D vaccinated NHPs (*n* = 4; 1–18 months post-vaccination, pooled serum) [81]. Of the vaccinees, 22 out of 23 participants’ serum and all 4 NHPS’ serum as a primary antibody could detect YFV-17D NS1 as measured by western blotting, indicating the presence of anti-NS1 antibodies in the serum. This finding that anti-NS1 antibodies could be detected several years after YFV-17D vaccination warrants further investigation as to the durability of these antibodies. Most recently, work by Mantel *et al.* 2024 comparing immunogenicity of YFV-17D in humans vs. NHPs demonstrated that anti-NS1 IgG antibodies were detectable in all tested humans (*n* = 20) and NHP serum (*n* = 18) at 21 and 28 days post-vaccination (range of >1 to 4 log EU/mL) [82]. This study used anti-NS1 IgG titers as a surrogate marker for viral replication after vaccination and did not analyze the NS1 antibody response further. Another study by Kalimuddin *et al.* 2025 demonstrated that all participants in a cohort of YFV-17D vaccinees (*n* = 16) had detectable anti-NS1 antibodies at 28 days post-vaccination [83]. Together these studies, despite the small cohort size, still provide evidence that anti-NS1 antibodies are present soon after YFV-17D vaccination. Further studies are needed to validate these findings in larger, more diverse vaccinee populations and identify what factors after vaccination regulate the production of this understudied antibody population.

Other evidence of the potential importance of anti-NS1 antibodies in providing protection from orthoflavivirus infection comes from the debate over the efficacy of current DENV vaccines. Dengvaxia(R) (CYD-TDV), produced by Sanofi, is a live attenuated vaccine platform of DENV1-4 chimeric viruses in a YFV-17D backbone engineered to expressed DENV structural proteins and YFV-17D non-structural proteins, including NS1 [84]. CYD-TDV vaccination found to be effective only in previously DENV infected individuals and was associated with elevated hospitalization incidence in naïve vaccinees, leading to the recommendation that only previously DENV exposed individuals should receive the vaccine [85]. Some argue that in addition to issues arising from cross-reactive non-neutralizing antibodies that may contribute to vaccine induced disease via antibody-dependent enhancement, the lack of DENV specific anti-NS1 antibodies also contributed to the limited protection conferred by CYD-TDV [86].The Takeda dengue vaccine, Qdenga (R) (TAK-003), is also a chimeric vaccine in a DENV2 backbone that expresses only DENV2 NS1, and in vitro evidence suggests that the DENV2 NS1 specific antibodies may contribute to protection against vascular leak in DENV infection [87]. The NIH designed dengue vaccine TV-003 incorporates DENV1, 3, and 4 NS1 in their LAV cocktail, and may elicit additional serotype specific NS1 antibodies compared to the other two vaccine platforms, although to date the relative titers of NS1 antibodies against NS1 in vaccinees have not been well characterized [88].

Nevertheless, with the growing body of evidence that NS1 is important in the pathogenicity of several orthoflaviviruses, we argue that the lack of research on the most successful orthoflavivirus YFV-17D’s vaccine’s ability to induce anti-NS1 antibodies illustrates a clear gap in our knowledge of YFV-17D mechanisms of immunity. As described in the following sections below, YFV specific anti-NS1 antibodies have the potential to be an influential aspect of adaptive immunity against YFV infections and thus warrants further study in both models of YFV-17D vaccination and YFV natural infection. These gaps in knowledge regarding mechanisms of protection mediated against YFV NS1 may become more important in the changing dynamics of vector transmission due to changes in land usage, climate, and socioeconomic shifts in South America and Africa which could result in increased YFV outbreaks amidst the challenging demand of YFV-17D vaccine production [89, 90].

### YFV anti-NS1 antibodies can provide protection in vivo

While the direct role of YFV NS1 mediated pathogenesis is still unclear – there have been several studies that demonstrate protection elicited by anti-YFV NS1 antibodies. The first described study demonstrated protection in Swiss CD1 mice through passive administration of anti-NS1 mAbs or vaccination with YFV-17D derived NS1 (formerly called gp48) against a lethal YFV-17D encephalitic challenge model [91]. The investigators further found that only the anti-NS1 mAbs that could induce complement fixation could provide protection in this model due to the ability to induce complement mediated cell lysis of infected cells. Notably, because only NS1 mAbs or vaccination with NS1 were used to induce antibodies, neutralizing antibodies were absent in this study, indicating that protection could be achieved through YFV anti-NS1 antibodies alone. Evidence of anti-NS1 mediated protection was also illustrated by Putnak *et al.* when BALB/c mice were intraperitoneally vaccinated with a recombinant vaccinia virus encoding the YFV-17D gene from NS1 to partial NS3 and then 3-weeks later intracerebrally challenged with 4 × 10^5^ pfu of YFV-17D [92]. They found that 10 out of 20 recombinant vaccina virus vaccinated mice survived compared to none of the 20 mice vaccinated with wild type vaccinia virus.

As noted above, YFV infection primarily damages the liver in humans. Animal models that better recapitulate this disease phenotype include rhesus macaques and Syrian golden hamsters [93]. However, for Syrian golden hamsters there are only two adapted strains of YFV that recapitulate disease, the Asibi and Jimenez strains [94]. Thus, most animal models of YFV infection have relied on rhesus macaques [26, 95]. Using this rhesus macaque animal model, Schlesinger *et al.* in 1986 first demonstrated that vaccination with three doses of YFV-17D derived NS1 (100 µg first dose; 50 µg for boosters) with alum provided protection from death in 4 out of 5 of macaques that were challenged with the YFV Dakar 1279 strain [96]. However, in this study 2 of the 5 macaques that survived had low levels of neutralizing antibodies by plaque reduction neutralization test (PRNT), which may confound the interpretation of these data.

In an effort to eliminate the presence of other YFV viral proteins present in NS1 vaccine preparations to avoid the potential confounding in Schlesinger’s study indicated by the PRNT results, Cane *et al.* 1988 expressed an NS1-β-galactosidase fusion protein in E.coli and used this to vaccinate T0 mice [97]. However, this fragmented NS1 construct spanned just a portion of the wing domain and provided only partial protection against less virulent YFV strains – indicating, based on the earlier studies above, that the full NS1 protein likely provides better immunogenicity. Further studies have identified that the binding or interference of regions of dengue NS1 β-ladder and wing domains are critical for inhibiting NS1 mediated endothelial permeability through anti-NS1 antibodies and are likely why there was reduced protection in the Cane study [98, 99].

More recent studies that have looked at non-neutralizing antibody mediated protection in animal models come from various studies that utilize the YFV-17D strain as a backbone for the formulation of chimeric viruses with other orthoflaviviruses. Mishra et al. 2020 developed a YFV vaccine based on YFV-17D that expressed the prM and Env genes of the JEV SA14-14-2 vaccine strain, termed JE-CVax [100]. AG129 mice vaccinated with 10^4^ PFU of JE-CVax demonstrated 97% protection (35/36 mice) against lethal YFV-17D challenge at 28 days post-vaccination while all placebo (*n* = 38) mice succumbed to infection. Serum from JE-CVax vaccinated mice demonstrate anti-NS1 antibody presence and serum could induce antibody dependent cellular cytotoxicity when incubated with YFV-17D mCherry expressing HEK293T cells and Jurkat cells. However, they did not specify what antibody population mediated this cytotoxic response, although given the surface expression of YFV NS1 this is likely due to anti-NS1 antibodies.

Another study by Kum *et al.* 2020 generated a YFV-17D backbone with ZIKV prM/Env genes to vaccinate AG129 mice (10^4^ PFU of YF-ZIKprM/Env; *n* = 9) and challenged with 10^3^ PFU of YFV-17D, resulting in complete protection in the vaccinated compared to no survival in sham vaccinated mice (*n* = 10) [101]. However, in this study passive serum transfer did not elicit protection in AG129 mice, indicating that for this YFV-17D chimeric vaccine non-neutralizing antibodies play a minor role compared to cellular immunity for protection. Together, these studies highlight the need to further study how anti-NS1 antibodies may or may not provide protection in animal and human models.

### Importance of anti-NS1 antibody Fc functionality

While most protective studies of antibodies focus on the interaction of the Fab (antibody epitope binding fragment) with its antigen – there is growing evidence within the orthoflavivirus field that the invariant Fc (crystallizable fragment) domain’s effector functions are also crucial in providing protection. Antibody Fc domains have a few features that influence their binding to classical Fc receptors (FcRs) or the non-classical c-type lectin receptors (CLRs) such as the hinge regions flexibility, disulfide bonds, and glycosylation sites [102]. In addition to potentially altering the binding capacity of the Fab domain to its antigen, the Fc domain once bound to its receptors can induce a variety of effector functions including complement activation, phagocytosis (neutrophils/macrophages), and degranulation (Natural killer cells, mast cells, basophils, and eosinophils). Antibodies are isotyped by their Fc (IgG, IgM, IgA, IgE, IgD) which can be further subclassed into IgG1, IgG2, IgG3, IgG4, IgA1, and IgA2. In a study by Dias Jr *et al.* 2022 using a longitudinal Nicaraguan pediatric cohort of DENV immune children, they found that anti-DENV Env and NS1 antibodies that induced complement deposition were higher in inapparent secondary DENV infected individuals compared to symptomatic secondary DENV infected individuals, indicating that the Fc functionality of their antibodies were important in providing protection against infection [103].

While both anti-Env and anti-NS1 antibodies can bind to infected cells, Schlesinger *et al.* in 1990 demonstrated that anti-NS1 antibodies were vastly better at inducing complement mediated cytolysis in vitro, likely due to the elevated NS1 on the surface of infected cells [42]. This ability of anti-NS1 antibodies to induce complement mediated cell lysis highlights the importance of Fc mediated effector functions in the mechanism of protection for anti-NS1 antibodies. The importance of Fc effector functionality was further detailed in a follow up study by Schlesinger et al. 1993 that saw reductions in anti-NS1 antibody mediated protection when mouse mAb Fc portions were switched from IgG2a to IgG2B or IgG1 or removed entirely leaving just the Fab portion in a YFV-17D encephalitic challenge model [104]. IgG2a is described to most effectively induce antibody dependent cell cytotoxicity in mice, while IgG2b and IgG1 are less capable [105]. Together, these studies indicate that a potential functional requirement for anti-NS1 antibody mediated protection is determined by Fc effector function capacity. Since Schlesinger’s initial report in 1985, there have been numerous studies that illustrate the protective capacity of anti-NS1 antibodies for a variety of other orthoflaviviruses with varying degrees of importance of Fc effector function capacity [9–11, 106–109].

Finally, in a study by Medina-Magues *et al.* 2023 they generated a mRNA vaccine that encodes either the YFV-17D prM/Env or NS1 gene, all encapsulated in a lipid nanoparticle [110]. They vaccinated A129 mice with the NS1 mRNA-LNP and passively transferred these anti-NS1 antibodies into another set of A129 mice prior to lethal YFV Asibi challenge (10^5^ PFU), resulting in viremia but also protection against severe disease in 4 out of 5 mice. Interestingly, when they passively transferred anti-NS1 antibodies from NS1 mRNA-LNP vaccinated rhesus macaques into mice these antibodies no longer provided protection which the authors suggest could be due to differences in antibody Fc effector functionality across these species. What the Fc effector profile is like in humans after YFV-17D vaccination and whether this is important is currently unknown.

### YFV anti-NS1 antibodies can be cross-reactive

The potential cross-reactivity of anti-NS1 antibodies against other YFV genotype strains and even other orthoflaviviruses is an area of interest that could enhance YFV antibody mediated protection. Gould *et al.* 1985 created a panel of mouse mAbs directed against Env and NS1 (termed 48 K at the time) by vaccinating BALB/c mice with YFV-17D or the French neurotropic vaccine (FNV) virus (FNV) [111]. They tested their 13 anti-48 K monoclonal antibodies for cross-reactivity via immunofluorescence staining of Vero cells infected with a panel of 34 YFV viruses that included: 10 different YFV-17D virus stocks isolated from a variety of sources, YFV17DD vaccine from Brazil, French Neurotropic YFV FNV, Asibi, YFV strains isolated from Brazil (sources include monkeys, humans, and Haemagogus), YFV strains isolated from humans from Senegal/Colombia, and YFV strains isolated from monkeys from Trinidad. They found high cross-reactivity for all of their anti-48 K mAbs against all viruses in their panel, indicating that anti-NS1 antibodies derived from YFV-17D vaccination have the potential for wide antibody breadth against YFV viral variants. In a follow-up to this paper, they intraperitoneally inoculated 12 of these mAbs each into T0 mice that were then intracerebrally challenged with 100 LD_50_ of YFV-17D derived strain 24 h later [112]. 8 of the 12 mAbs exhibited at least 50% protection against the YFV-17D strain, however, they stated that there was no protection when using other YFV strains although they do not state which strains were used. However, in a separate study by Deubel et al. 1987, YFV-17D derived monoclonal anti-NS1 antibodies immunoprecipitated NS1 proteins from YFV isolates from West Africa (Ivory Coast-Burkina Faso and Senegal) but were unable to precipitate NS1 using isolates from South America and Central Africa indicating a lack of cross-reactivity from these anti-NS1 antibodies [113].

In terms of cross reactivity of YFV specific anti-NS1 antibodies with other orthoflaviviruses, the data to date are less convincing. After generating anti-YFV NS1 antibodies by vaccinating rabbits (NS1 wing domain peptide 91–105 AA), and guinea pigs (wing domain peptide 128–143 AA or β-ladder domain peptide 188–202 AA) there was no cross-reactivity of these serum antibodies as measured through indirect immunofluorescence assay by Vero cells infected with DENV1-4, Tick borne encephalitis (TBEV), WNV, or JEV [58]. In another study, hybridomas derived from YFV-17D vaccinated BALB/c mice spleens were used to generate a panel of mAbs directed against Env and NS1 (called gp48 or NV3 at the time) [114]. The anti-NS1 antibodies could induce complement fixation against the YFV-17D and Asibi strains, and in a follow-up study they demonstrated orthoflavivirus cross-reactivity via immunofluorescence staining with the other orthoflaviviruses Koutango and Banzi viruses [115]. For human derived anti-NS1 antibodies, Chao et al. 2015 found minimal cross-reactivity of YFV-17D vaccinee serum against WNV NS1 (3/10; IgM) and DENV2, DENV3 NS1 (1/10; IgG) [116]. However, this study did not specify whether these vaccinees had any evidence of other orthoflavivirus infections that may influence these results. Together, the current data on YFV specific anti-NS1 cross-reactivity with other orthoflaviviruses of medical importance is not well described.

### YFV NS1 and T cells

YFV-17D vaccination induces T cell responses that are not as potent as compared to antibody responses. In a study by Monath *et al.* in 2002 they vaccinated YFV-17D immune individuals (*n* = 6) with ChimeriVax-JE – a chimeric virus that contains YFV-17D capsid/non-structural proteins but JEV prM/Env proteins [117]. ChimeriVax-JE “challenge” for those that were previously YFV-17D vaccinated 10–12 months prior inhibits the protection offered by their neutralizing antibodies since the YFV envelope protein is absent – however, the remaining T cell responses did not provide protection as evident by the detectable levels of viremia after challenge. Notably, this sample size is quite small, and it is unclear if these findings recapitulate the larger population of YFV-17D vaccinees. A more recent study by Kalimuddin et al. 2025 used human cohorts of individuals vaccinated with YFV-17D (*n* = 16) or JEV/17D (*n* = 17) and then “challenged” with the opposite vaccine 28 days later [83]. In this study there was no correlation between pre-challenge anti-NS1 antibody titers and viral RNA levels post-challenge, with the study further providing evidence that T cell specific responses were the main drivers of protection in the absence of neutralizing antibodies. However, the time from vaccination to challenge was only 28 days, so it is likely that anti-NS1 antibody responses were not fully developed at this time.

Nonetheless, in addition to the anti-NS1 antibody mediated immunity discussed above, there has been additional work that has characterized CD8 and CD4 T cell immunity against YFV NS1 after YFV-17D vaccination. Using a library of 870 peptides that spans the entire YFV-17D genome (15 mer peptides that overlap by 11 amino acids), Stryhn et al. 2020 identified 288 total YFV specific CD4 + and CD8 + T cell epitopes within their vaccinee cohort (*n* = 210) [118]. Of these epitopes, 18 for CD4 + T cells and 12 for CD8 + T cells were within the NS1 protein spanning across all three domains. This low number of NS1 specific epitopes recognized by CD4 + T cells is also described in other studies that indicate that the highest epitope densities are located in the YFV-17D Capsid, Envelope, NS2A, and NS3 proteins [83, 119].

Effector CD8 + T cell responses are influenced by the magnitude of viral load after YFV1 7D vaccination [120]. Co *et al.* 2002 created YFV specific CD8 + T cell lines from YFV-17D vaccinees and identified one cell line that recognized the 20-mer corresponding to residues 351–370 of YFV NS1 within the β-ladder domain that were HLA-B35 restricted [121]. In another study Blom et al. 2013 identified amino acids 945 − 933 (VYMDAVFEY) within the NS1 β-ladder domain as an epitope for CD8 + T cells within their cohort of 13 YFV-17D vaccinees that they further characterized in a cohort of 21 vaccinees and found that T cell responses against this epitope were transient, peaking at 15 days post-vaccination (dpv) but undetectable by 19 dpv [122]. While other studies have demonstrated the longevity of CD8 + T cells after vaccination, such as the detection of NS4B specific T cells in individuals that are 25 years post-YFV-17D vaccination, based on the Blom study it does not seem that NS1 specific T cells may be as durable [123].

## Conclusions

Research on mechanisms of protection against YFV induced by the widely successful vaccine YFV-17D can serve as a model to better our understanding what facets of adaptive immunity are critical in mounting effective protection. The findings from work with YFV-17D could be expanded for the other orthoflaviviruses for which vaccine development has been historically quite difficult. Here we have discussed the current state of knowledge regarding YFV NS1 with some key points to summarize. YFV NS1 seems to work similarly to other orthoflavivirus NS1 proteins using in vitro and in vivo models and is likely involved in pathogenesis. Antibodies directed against YFV NS1 can be protective as shown in mouse and NHP models and protection may be influenced by its Fc effector function capacity, which is particularly important given the non-neutralizing effect of these antibodies. YFV-17D, while widely used for decades in YFV prevention, has surprisingly little info on anti-NS1 mediated responses but some data suggests that there are anti-NS1 antibodies and T cells (CD4 and CD8) that are produced after vaccination.

This review highlights remaining gaps in our understanding of YFV NS1 and points to possible directions of future work. The role of YFV NS1 in the mosquito is particularly understudied and is necessary to further our understanding of host-vector YFV transmission dynamics. There is a critical need to study YFV-17D adaptive immunity (humoral and cellular) with respect to NS1 in larger cohorts to more rigorously test the functionality and durability of memory responses to NS1, as the current literature focuses primarily on neutralizing antibodies. These future cohort studies would benefit from the inclusion of participants from endemic areas with an emphasis on studying YFV natural infection which could enhance our understanding of NS1 mechanisms of pathogenesis. Considering the recurring YFV-17D vaccine shortages it is possible that research into the development of anti-NS1 antibody therapeutics could potentially aid in outbreaks as a means of providing a secondary level of protection in addition to fractional dosing. These therapeutic anti-NS1 antibodies would not be expected to induce antibody dependent enhancement which occurs for cross-reactive antibodies directed against the Env protein. Ultimately, there remain a lot of unknowns regarding YFV NS1 that may provide answers that are translatable to other medically relevant orthoflaviviruses.

## Supplementary Information

Supplementary Material 1.

## Data Availability

No datasets were generated or analysed during the current study.

## Abbreviations

YFV: Yellow fever virus
DENV: Dengue virus
WNV: West nile virus
JEV: Japanese encephalitis virus
ZIKV: Zika virus
TBEV: Tick borne encephalitis virus
YFV-17D: Yellow fever 17D vaccine strain
NS1: Non-structural protein 1
sNS1: secreted non-structural protein 1
Env: Envelope protein
WHO: World health organization
NAbs: Neutralizing antibodies
HA: Hemagglutinin
ER: Endoplasmic reticulum
mAbs: monoclonal antibodies
ROS: Reactive oxygen species
GAGS: Glycosaminoglycans
TLR4: Toll-like receptor 4
TEER: Trans-endothelial electrical resistance
HLSEC: Human liver sinusoidal endothelial cells
NHP: Non-human primate
CYD-TDV: Dengvaxia vaccine (Sanofi)
TAK-003: Dengue vaccine (Takeda)
LAV: Live attenuated vaccine
LNP: Lipid nanoparticle
gp48: glycoprotein 48 kda (former name for NS1)
PFU: Plaque forming units
PRNT: Plaque reduction neutralization test
Fab: Fragment antibody binding
Fc: crystallizable fragment antibody
Dpv: Days post-vaccination
