# Supplementary material for: Endothelial leak and knowledge gaps: yellow fever virus non-structural protein 1 and the antibodies that bind it
Source: Virol J. 2025 Oct 21;22:338. doi: 10.1186/s12985-025-02955-9 (PMC12538857; doi:10.1186/s12985-025-02955-9)
Supplement: Supplementary file 1 — Supplementary Material 1. [file 12985_2025_2955_MOESM1_ESM.docx]

| **Accession Number** | **Isolation Source** | **Location** | **Date** | **YFV Genotype** |
| --- | --- | --- | --- | --- |
| AY968064 | Human | Angola | 1971 | Angola |
| PQ441951 | Human | Sudan | 2003 | East Africa |
| AY968065 | Human | Uganda | 1948 | East Africa |
| DQ235229 |  | Ethiopia |  | East Central Africa |
| JN620362 | Human | Uganda | 2010 | East Central Africa |
| JX898869 | Aedes africanus | Cote d’Ivoire | 1973 | West Africa I |
| U54798 | Human | Cote d’Ivoire | 1982 | West Africa I |
| NC_002031 | YFV 17D vaccine | NA | 1985 | West Africa I |
| MZ595203 | Human | Senegal | 2020 | West Africa I |
| KF769016 | Human (Asibi) | Ghana | 1927 | West Africa I |
| ON323054 | Human | Nigeria | 2020 | West Africa II |
| AF094612 | Haemogogus spegazzini | Trinidad | 1979 | West Africa II |
| MG051218 | Human (French neurotropic virus) | NA | 2019 | West Africa II |
| JX898873 | Aedes furcifer | Senegal | 2000 | West Africa II |
| MZ595193 | Mosquito | Senegal | 2020 | West Africa II |
| JX898870 | Aedes furcifer | Senegal | 1996 | West Africa II |
| U21056 | Human (French viscerotropic virus) | Senegal | 1927 | West Africa II |
| MW158345 | Haemogogus janthinomys | Trinidad | 1988 | South America I |
| MW158347 | Alouatta | Trinidad | 1989 | South America I |
| MW158338 | Sentinel monkey | Brazil | 1955 | South America I |
| MW158340 | Saguinus midas | Brazil | 1968 | South America I |
| OR119828 | Human (Jimenez) | Panama | 1974 | South America I |
| KY861728 | Alouatta species | Brazil | 2008 | South America I |
| MW158344 | Haemogogus janthinomys | Brazil | 1985 | South America I |
| MW158343 | Haemogogus species | Brazil | 1973 | South America I |
| ON022700 | Alouatta species | Brazil | 2016 | South America I |
| MT497520 | Sapajus species | Brazil | 2016 | South America I |
| MZ712127 | Alouatta guariba clamitans | Brazil | 2021 | South America I |
| JF912187 | Human | Brazil | 2000 | South America I |
| JF912185 | Sabethes species | Brazil | 1992 | South America I |
| JF912186 | Human | Brazil | 1994 | South America I |
| JF912184 | Human | Brazil | 1987 | South America I |
| JF912179 | Haemogogus | Brazil | 1980 | South America I |
| JF912183 | Human | Brazil | 1984 | South America I |
| PQ879118 | Alouatta species | Brazil | 2024 | South America I |
| OQ572696 | Sabethes chloropterus | Brazil | 2021 | South America I |
| ON022696 | Alouatta | Brazil | 2019 | South America I |
| ON022652 | Non-human primate | Brazil | 2017 | South America I |
| ON022611 | Non-human primate | Brazil | 2017 | South America I |
| ON022475 | Human | Brazil | 2018 | South America I |
| MK533792 | Alouatta guariba clamitans | Brazil | 2019 | South America I |
| MH193173 | Alouatta species | Brazil | 2018 | South America I |
| MF347613 | Human | Suriname | 2017 | South America I |
| KM388817 | Alouatta seniculus | Venezuela | 2004 | South America I |
| HM582851 | Alouatta seniculus | Trinidad | 2009 | South America I |
| MW158359 | Human | Peru | 1998 | South America II |
| PP477075 | Human | Colombia | 2023 | South America II |
| JF912181 | Human | Brazil | 1983 | South America II |
| MW158361 | Human | Peru | 1995 | South America II |
| MF004382 | Human | Bolivia | 1999 | South America II |
| PQ181571 | Human | Peru | 1998 | South America II |

**Supplementary Table 1.** Accession numbers used for the YFV NS1 sequence alignment (n=51) with information regarding isolation and genotype.


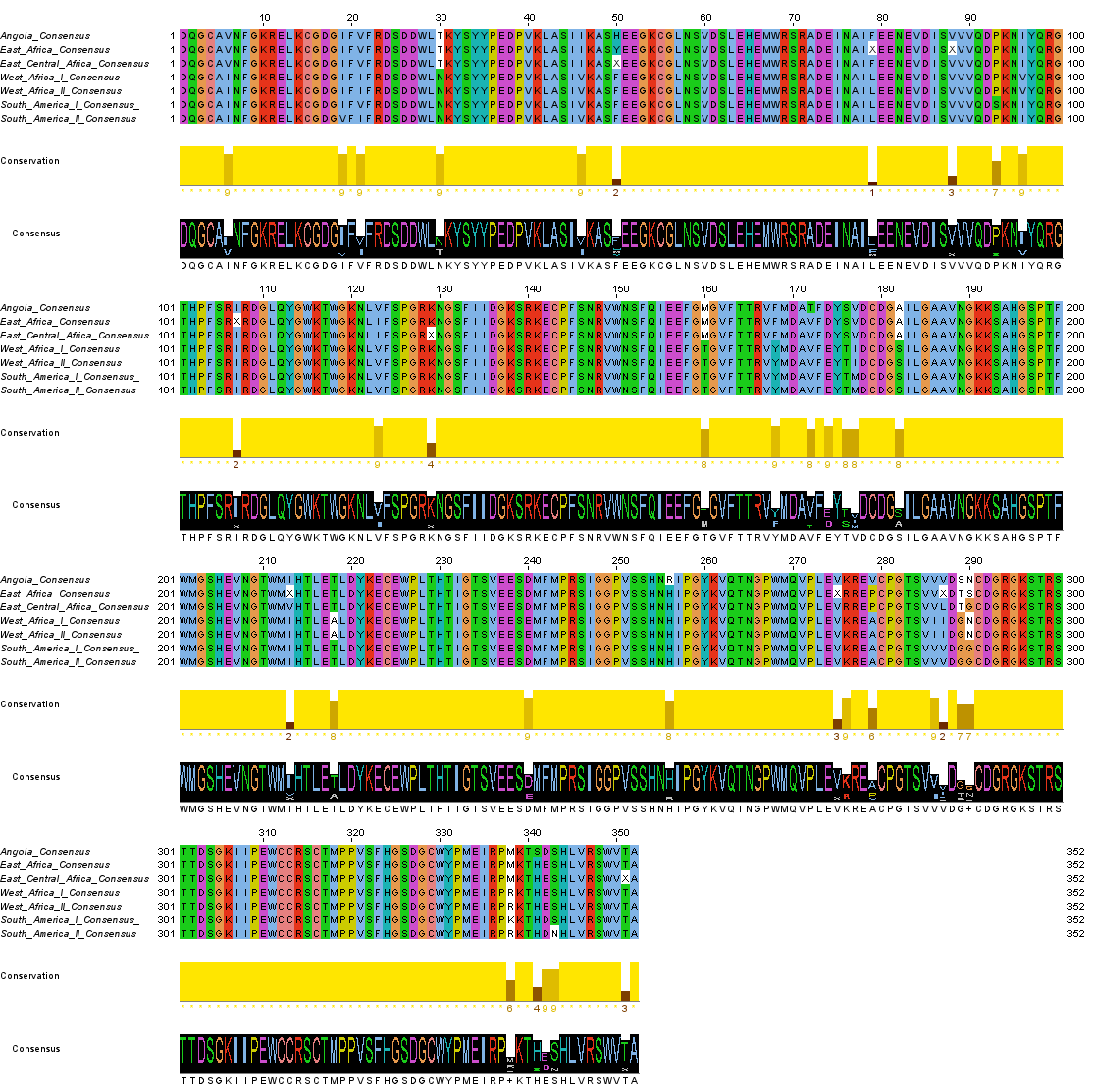


**Supplementary Fig 1**. Consensus sequences were generated using Clustal Omega alignment of sequences listed in supplementary table 1 using Geneious. For East Africa and East Central Africa genotypes only 2 sequences used, so differences between the two are indicated by an “X”, the AA for these are found in supplemental table 2. These 7 consensus sequences were then aligned using jalview. Amino acid color categories: blue is hydrophobic, red is positive charge, magenta is negative charge, green is polar, pink is cysteines, orange is glycines, yellow is prolines, cyan is aromatic, and white is unconserved. Conservation scores in yellow, with the star (*) representing the highest conservation score of 11. The colored bars above indicate domains: β-roll domain (blue, AA 1-29), discontinuous connector subdomain of wing (orange, AA 30-37 and 152-180), wing domain (yellow, AA 38-151), and β-ladder domain (red, AA 181-352). Glycosylation sites indicated by the green star (AA 130 and 208).

| **β-Roll Domain (AA 1 – 29)** | | |
| --- | --- | --- |
| **AA Differences** | **Strains** | **Genotypes** |
| I 6 V | AY968064 | Angola |
|  | PQ441951, AY968065 | EA |
|  | DQ235229, JN620362 | ECA |
|  | MW158340 | SA I |
| G 9 S | JF912179 | SA I |
| K 10 R | MF004382 | SA II |
| I 19 V | MW158359, JF912181, MW158361, PQ181571, MF004382 | SA II |
| V 21 I | JX898869, U54798, NC_002031, MZ595203, AY640589 | WA I |
|  | ON323054, AF094612, MW249092, JX898873, MZ595193, JX898870, U21056 | WA II |
|  | MW158338, MW158340, OR119828, MW158344, MW158343 | SA I |
|  | MW158359, PP477075, JF912181, MW158361, MF004382, PQ181571 | SA II |

| **Wing Domain (AA 30 – 180)** | | |
| --- | --- | --- |
| **AA Differences** | **Strains** | **Genotypes** |
| N 30 T | AY968064 | Angola |
|  | PQ441951, AY968065 | EA |
|  | DQ235229, JN620362 | ECA |
| N 30 S | JF912186, JF912184, JF912179, JF912183 | SA I |
| N 30 D | PQ879118, OQ572696 | SA I |
| V 46 I | AY968064 | Angola |
|  | PQ441951, AY968065 | EA |
|  | DQ235229, JN620362 | ECA |
| F 50 H | AY968064 | Angola |
|  | DQ235229 | ECA |
| F 50 Y | PQ441951, AY968065 | EA |
|  | JN620362 | ECA |
| E 51 D | MK533792 | SA I |
| E 64 D | U54798 | WA I |
| L 79 F | AY968064 | Angola |
|  | AY968065 | EA |
|  | DQ235229, JN620362 | ECA |
|  | OR700702 | WA II |
|  | NC_002031 | WA I |
|  | PP477075 | SA II |
| V 88 I | AY968065 | EA |
| D 92 N | ON022700 | SA I |
| S 93 P | AY968064 | Angola |
|  | PQ441951, AY968065 | EA |
|  | DQ235229, JN620362 | ECA |
|  | JX898869, U54798, NC_002031, MZ595203, AY640589 | WA I |
|  | ON323054, AF094612, MW249092, JX898873, MZ595193, JX898870, U21056 | WA II |
|  | MW158359, PP477075, JF912181, MW158361, MF004382, PQ181571 | SA II |
| I 96 V | JX898869, U54798, NC_002031, MZ595203, AY640589 | WA I |
|  | ON323054, AF094612, MW249092, JX898873, MZ595193, JX898870, U21056 | WA II |
| P 103 S | JF912184 | SA I |
| I 107 V | PQ441951 | EA |
|  | ON022696 | SA I |
| K 120 R | PP477075 | SA II |
| N 121 S | JX898873 | WA II |
|  | MW158359 | SA II |
| V 123 I | PQ441951, AY968065 | EA |
|  | DQ235229, JN620362 | ECA |
| K 129 R | JN620362 | ECA |
| T 160 M | AY968064 | Angola |
|  | PQ441951, AY968065 | EA |
|  | DQ235229, JN620362 | ECA |
| Y 168 F | AY968064 | Angola |
|  | PQ441951, AY968065 | EA |
|  | DQ235229, JN620362 | ECA |
| V 172 T | AY968064 | Angola |
| E 174 D | AY968064 | Angola |
|  | PQ441951, AY968065 | EA |
|  | DQ235229, JN620362 | ECA |
| Y 175 H | MH193173 | SA I |
| T 176 S | AY968064 | Angola |
|  | PQ441951, AY968065 | EA |
|  | DQ235229, JN620362 | ECA |
| M 177 V | AY968064 | Angola |
|  | PQ441951, AY968065 | EA |
|  | DQ235229, JN620362 | ECA |
| M 177 I | JX898869, U54798, NC_002031, MZ595203, AY640589 | WA I |
|  | ON323054, AF094612, MW249092, JX898873, MZ595193, JX898870, U21056 | WA II |

| **β-ladder Domain (AA 181 – 352)** | | |
| --- | --- | --- |
| **AA Differences** | **Strains** | **Genotypes** |
| S 182 A | AY968064 | Angola |
|  | PQ441951, AY968065 | EA |
|  | DQ235229, JN620362 | ECA |
| S 193 G | ON323054 | WA II |
| A 194 T | ON323054 | WA II |
| F 200 S | MZ595193 | WA II |
| H 205 N | JX898870 | WA II |
| M 212 V | OR119828 | SA |
| I 213 V | PQ441951 | EA |
|  | DQ235229, JN620362 | ECA |
| I 213 M | AY968065 | EA |
| T 218 A | NC_002031, MZ595203, AY640589 | WA I |
|  | ON323054, AF094612, MW249092, JX898873, MZ595193, JX898870, U21056 | WA II |
| D 220 N | MF347613 | SA I |
| L 228 P | U21056 | WA II |
| S 235 P | MW158347 | SA I |
| V 236 I | MZ595203 | WA I |
|  | MZ595193 | WA II |
| S 239 G | ON022652 | SA I |
| D 240 E | NC_002031, MZ595203, AY640589 | WA I |
|  | ON323054, AF094612, MW249092, JX898873, MZ595193, JX898870, U21056 | WA II |
| D 240 N | MW158359 | SA II |
| H 256 R | AY968064 | Angola |
| T 264 I | MW158359 | SA II |
| V 275 I | PQ441951 | EA |
| K 276 R | PQ441951, AY968065 | EA |
|  | DQ235229, JN620362 | ECA |
|  | MZ595203 | WA I |
|  | JX898873, MZ595193, JX898870 | WA II |
|  | HM582851 | SA I |
| A 279 V | AY968064 | Angola |
| A 279 P | PQ441951, AY968065 | EA |
|  | DQ235229, JN620362 | ECA |
| S 284 N | KY861728, MW158344, MW158343 | SA I |
| V 286 I | JX898869, U54798, NC_002031, MZ595203, AY640589 | WA I |
|  | ON323054, AF094612, MW249092, JX898873, MZ595193, JX898870, U21056 | WA II |
| V 286 A | KM388817 | SA I |
| V 287 L | PQ441951 | EA |
|  | DQ235229, JN620362 | ECA |
| V 287 I | JX898869, U54798, NC_002031, MZ595203, AY640589 | WA I |
|  | ON323054, AF094612, MW249092, JX898873, MZ595193, JX898870, U21056 | WA II |
| G 289 S | AY968064 | Angola |
| G 289 T | PQ441951, AY968065 | EA |
|  | DQ235229, JN620362 | ECA |
| G 290 N | AY968064 | Angola |
|  | JX898869, U54798, NC_002031, MZ595203, AY640589 | WA I |
|  | ON323054, AF094612, MW249092, JX898873, MZ595193, JX898870, U21056 | WA II |
| G 290 S | PQ441951, AY968065 | EA |
|  | PQ879118, ON022611 | SA I |
| G 290 D | MW158338 | SA I |
|  | MW158361 | SA II |
| G 305 R | ON323054 | WA II |
| I 307 V | NC_002031 | WA I |
|  | ON022700, MT497520 | SA I |
| I 308 V | ON022475 | SA I |
| S 315 A | MW158343 | SA I |
| H 324 R | MW158361 | SA II |
| S 326 N | JX898869, U54798 | WA I |
| P 332 S | MW158345, MW158347, KY861728, JF912187 | SA I |
| K 338 M | AY968064 | Angola |
|  | PQ441951, AY968065 | EA |
|  | DQ235229, JN620362 | ECA |
|  | AF094612. | WA II |
| K 338 R | JX898869, U54798, NC_002031, MZ595203, AY640589 | WA I |
|  | ON323054, MW249092, JX898873, MZ595193, JX898870, U21056 | WA II |
|  | MW158345, JF912185 | SA I |
|  | MW158359, PP477075, JF912181, MW158361, MF004382, PQ181571 | SA II |
| K 339 N | MW158359 | SA II |
| H 341 S | AY968064 | Angola |
| D 342 E | PQ441951, AY968065 | EA |
|  | DQ235229, JN620362 | ECA |
|  | JX898869, U54798, NC_002031, MZ595203, AY640589 | WA I |
|  | ON323054, AF094612, MW249092, JX898873, MZ595193, JX898870, U21056 | WA II |
|  | MW158359 | SA II |
| S 343 N | MZ712127 | SA I |
|  | MW158359, JF912181, MW158361, MF004382, PQ181571 | SA II |
| T 351 A | DQ235229 | ECA |

**Supplementary Table 2**. Amino acid differences across the 7 YFV genotypes by domain compared to a YFV consensus sequence. YFV strains across the 7 genotypes were selected for NS1 sequence alignment (n=51) to represent the diversity of YFV NS1. Amino acid (AA) changes are compared to the consensus sequence. Genotype abbreviations are: East Africa (EA), East Central Africa (ECA), West Africa I (WA I), West Africa II (WA II), South America I (SA I), and South America II (SA II). YFV strains are listed by their accession number, which is detailed in supplementary table 1.
